# How Meaningful is the Elite Quality Index Ranking?

**DOI:** 10.1007/s11205-021-02841-1

**Published:** 2022-02-16

**Authors:** Céline Diebold

**Affiliations:** Institute of Economics (FGN-HSG), University of St. Gallen, St. Gallen, Switzerland

**Keywords:** Index, Ranking, Elite quality, Uncertainty and sensitivity analysis

## Abstract

The Elite Quality Index (EQx) attempts to measure the propensity of elites—on aggregate—to create value, rather than to rent seek. The index has attracted worldwide media and press attention. In their articles, journalists have based their analyses primarily on their own countries’ position in the EQx ranking. But how meaningful is the EQx ranking? How do the uncertainties underlying some of the assumptions made in the index propagate to the country rankings? We conduct a global uncertainty and sensitivity analysis (UA and SA) of the EQx and compute Sobol’ first and total order sensitivity indices using state of the art estimators, in order to scrutinise the implications of index assumptions and assess the reliability of the EQx ranking. The UA suggests that the EQx ranking of 2021 (EQx2021) is largely stable for the top 50 countries, but exhibits considerable uncertainties especially for middle and lower performing countries. The SA highlights the handling of missing data, the normalisation process and the weighting scheme as most important methodological choices, while the largest potential for improvement is observed in how raw missing indicator data is handled.

## Introduction

The Elite Quality Index (Casas et al., 2020; Casas & Cozzi, 2020, 2021), or EQx, is a recently introduced political economy index that attempts to measure the novel concept of elite quality (Casas, forthcoming 2022). Its analytical framework posits that the business models chosen and run by a country’s elites are an important determinant of sustainable economic and human development. This is because within a given setting, a country’s resources (human, natural, financial or knowledge-based) are allocated by those leaders and decision-makers with the strongest coordination capacity—elites. The EQx considers elites that run business models that create more value than they extract to be of high quality, while low quality elites operate business models that primarily extract value through rent seeking.

The publication of the EQx in 2020 and 2021 has attracted attention in the global media and press, with newspaper articles in the German *Handelsblatt* (Riecke, 2020, 2021), the Swiss *NZZ* (Casas et al., 2021b), the Russian *Forbes* (Caфpoнoвa, 2020; Лaндa, 2021a) and *Russia Today* (RT, 2020), and the *Indian Economic Times* (Banik & Cozzi, 2020), along with media and press in Angola (Ferreira, 2021), Argentina (Berensztein, 2020), Brazil (Ribeiro, 2020), China (Casas & Howell, 2021a, 2021b), Finland (Rønne, 2021), Portugal (Neves, 2020, 2021), Singapore (Casas et al., 2021a), Turkey (Ol, 2020), Ukraine (Лaндa, 2021b), the United States (Cowen, 2020) and Kazakhstan (Учёт, 2020). In these articles, journalists primarily base their analyses on their own countries’ position in the EQx ranking. This ranking emerges as an output of the underlying model, which constructs the index. However, when modelling an index, uncertainties arise at each step of the construction, for instance, regarding the selection of the indicators to be used, and the chosen normalisation, weighting and aggregation scheme. This raises the questions: how reliable and meaningful is the EQx ranking of 2021 (EQx2021)? And how do the uncertainties that underlie the index assumptions propagate to the country ranking?

These questions can be addressed through an Uncertainty and Sensitivity Analysis (UA and SA). The UA aims to quantify the overall uncertainty i.e., variation in the model output as a result of the uncertainties in the inputs, while the SA “is the study of how the uncertainty in the output of a model (…) can be apportioned to different sources of uncertainty in the model input” (Saltelli, 2002, p. 579). Hence, a SA allows for the assigning of the uncertainty in the output to specific input factors or a combination thereof. Since indices can be considered models (Saltelli, 2002, p. 580), a combination of UA and SA can “gauge the robustness of the composite indicator ranking, to increase its transparency, to identify which countries are favoured or weakened under certain assumptions and to help frame a debate around the index” (OECD, 2008, p. 117).

In an attempt to assess the implications of the uncertainties in the model used to construct the EQx, Casas et al. (2020) conduct several robustness tests to explore how the model output changes when one factor is varied and the rest remain fixed. While this represents a widely used approach, Saltelli et al. (2019, p. 31) illustrate the issues around applying such a ‘one at a time’ (OAT) approach, positing that it represents a local method of sensitivity analysis that is problematic when the model is of unknown linearity. This is because the OAT approach does not take account of potential interactions between factors of uncertainty. For instance, if a model is based on 10 uncertain input factors (in this case, index assumptions), investigating the impact of these factors OAT “leaves over 99.75% of the input space totally unexplored” (Saltelli et al., 2019, p. 31). This renders the OAT sensitivity analysis “perfunctory, unless the model is proven to be linear” (Saltelli et al., 2019, p. 31). Hence, when an exploring an index such as the EQx, a local UA and SA approach is not appropriate, since the model output, e.g., the score and ranking of a given country, are non-linear functions of the uncertain input factors (OECD, 2008, 118ff; Saisana et al., 2005, p. 310).

Saltelli et al. (2019, p. 37) systematically review SA practices and advise that: “Global approaches are requisite to performing a valid sensitivity analysis when models feature nonlinearities and interactions” (p. 31). Global UA and SA take account of the entire variation in the range of inputs and explore all sources of uncertainty simultaneously, in order to capture possible synergy effects among uncertain input factors (Iooss & Saltelli, 2015, p. 2; OECD, 2008, p. 118). In a comment published in *Nature* (Saltelli et al., 2020), this approach was listed as one of the five cornerstones of responsible mathematical modelling, with the multidisciplinary team of scientists arguing that: “these global uncertainty and sensitivity analyses are often not done. Anyone turning to a model for insight should demand that such analyses be conducted, and their results be described adequately and made accessible” (p.483). Furthermore, a global UA and SA is specifically suitable for assessing indices, and it is recommended by the OECD *Handbook on constructing composite indicators* (2008).

One such technique is the analysis of variance (ANOVA) of the model output (Saltelli et al., 2019, p. 31), which is most appropriate for the analysis of composite indicators. (Iooss & Saltelli, 2015, p. 3; Saisana et al., 2005, p. 311). This is due to several properties that this approach possesses (OECD, 2008, p. 121; Saisana et al., 2005, p. 312): firstly, it represents a ‘model-free’ method and is thus suited for the analysis of non-linear models; secondly, it explores the entire variation in the range of inputs as well as their interactions; and thirdly, it allows for the investigation of main effects (first-order) as well as interaction effects (higher-order) between the input factors. Variance-based techniques have been used for several renowned indices to assess their robustness to changes in the methodology. These include, for example, the United Nations Human Development Index (Aguña & Kovacevic, 2011; Kuc-Czarnecka, 2019), the United Nations Technology Achievement Index (Saisana et al., 2005), and the Environmental Performance Index (Papadimitriou et al., 2020; Saisana & Saltelli, 2008).

Among the available sensitivity indices, variance-based indices are regarded as the “gold standard” (Puy et al., 2021b, p. 2). In particular, the OECD (2008, p. 123) suggests the computation of Sobol’ indices. The search for estimators of Sobol’ indices, that are both efficient and robust, is currently an active field of research. One recommended estimator according to Puy et al. (2021b, p. 19), is the Azzini estimator, recently presented by Azzini et al. (2020), as it is efficient and robust to a wide range of practical situations. To the best of our knowledge, this paper provides the first application of the Azzini estimator of Sobol’ indices to assess the performance of an index.

Therefore, this paper sets out to conduct a UA and SA, where we generally follow the recommendations of the OECD’s *Handbook on constructing composite indicators* (2008). However, we use more recent, state of the art tools. Thus, this paper contributes to the literature in two main ways. Firstly, it assesses the reliability and meaningfulness of the EQx2021 ranking, highlighting the most important of the uncertain input factors, by applying a global, variance-based UA and SA. Secondly, it utilizes state of the art techniques, by applying the Azzini estimator.

The remainder of this paper is organised as follows. The following section reviews the relevant literature. Section 3 presents the methodology, and Sect. 4 the results of a variance-based UA and SA of the EQx2021. Finally, Sect. 5 offers a conclusion and outlines both the limitations of this study and potential avenues for future research.

## Literature

### Monte Carlo Approach to Evaluating Output Uncertainty

In order to evaluate output uncertainty, the OECD (2008, p. 117) suggests following the approach presented by Saisana et al. (2005), to conduct a global UA and SA based on a single Monte Carlo experiment, where the index is calculated numerous times based on randomly selected input factors. Generally, this approach imposes only few assumptions on the functional form of the model (Puy et al., 2021b, p. 2).

The input factors trigger which, for instance normalisation, weighting and aggregation schemes are adopted (out of a pre-determined number of alternatives). For each set of input factors, the model is evaluated, yielding a probability distribution function (pdf) of the model output. This provides the basis for the UA, where the output distribution is presented and characterised (Saisana et al., 2005, p. 310), for instance, though the use of some summary statistics (e.g. mean, median and variance) (Saltelli et al., 2019, p. 30). In the next step, the characteristics of this pdf (for instance variance, higher-order moments) can be estimated and analysed. This approach can serve as a “quality assurance tool” (Saltelli et al., 2019, p. 30), by ensuring a thorough analysis of the implications of index assumptions, and, importantly, potential synergistic effects (OECD, 2008, 118ff; Saisana et al., 2005, p. 310).

### Variance-based Sensitivity Analysis

The OECD (2008, p. 123) suggest using the method of Sobol (1990) for a variance-based SA. The method is based on the decomposition of the variance of the model output into the sum of the variances of the input factors, in increasing dimensionality (also called functional ANOVA, or FANOVA (Prieur & Tarantola, 2015, p. 2)). That is, the functional decomposition of the variance $V$ of model output $Y$ based on a set of independent input factors ${X}_{i}, i=1,\dots k$ can be written as:

1 $$ V\left( Y \right) = \mathop \sum \limits_{i} V_{i} + \mathop \sum \limits_{i} \mathop \sum \limits_{j > i} V_{ij} + \ldots + V_{12 \ldots .k} $$

where $V\left(Y\right)$ is the unconditional variance of Y, when all input factors ${X}_{i}$ are allowed to vary. Furthermore, ${V}_{i}=V\left[{\mathbb{E}}\left(Y|{X}_{i}\right)\right]$ where ${\mathbb{E}}\left(Y|{X}_{i}\right)$ is the mean of $Y$ when one factor is fixed, and ${V}_{ij}= V\left[{\mathbb{E}}\left(Y|{X}_{i},{X}_{j}\right)\right]-{V}_{i}-{V}_{j}$ and so on for higher-order interactions.

Dividing each term in Eq. 1 by the unconditional model output variance $V\left(Y\right)$ yields the so-called *variance-based sensitivity indices* or *Sobol’ indices* (Iooss & Saltelli, 2015, p. 9; Pianosi et al., 2016, p. 222; Saisana et al., 2005, p. 311; Sobol, 1990):

2 $$ S_{i} = \frac{{V_{i} }}{V\left( Y \right)}, S_{ij} = \frac{{V_{ij} }}{V\left( Y \right)}, \ldots $$

These sensitivity indices indicate the share of variance of the model output $Y$ due to the uncertainty of a single input factor, a pair of input factors, as well as higher-order terms. The *first-order sensitivity index* ${S}_{i}$ is “the most prevalent example of a global measure” and indicates “the expected fractional reduction in the variance of y that would be achieved if factor ${x}_{i}$ could be fixed” (Saltelli et al., 2019, p. 31). Hence, if ${S}_{i}$ is equal to 1, the variance in the model output is entirely driven by the input factor $i$. In this case, this input factor uniquely determines the model output.

The calculation of higher-order sensitivity indices can become computationally expensive (Iooss & Saltelli, 2015, p. 9). In consequence, to provide a “good description of model sensitivities” OECD (2008, p. 123), Saisana et al. (2005, p. 311) and the OECD recommend considering the first-order sensitivity index ${S}_{i}$, as well as the *total effect sensitivity index* ${S}_{Ti}$, which was first proposed by Homma & Saltelli (1996):

3 $$ S_{Ti} = S_{i} + \mathop \sum \limits_{i > j} S_{ij} + \mathop \sum \limits_{j \ne i, k \ne i, j < k} S_{ijk} + \ldots $$

For instance, for a model with $k=3$ input factors, the total sensitivity index for the first input factor would be:

4 $$ S_{T1} = S_{1} + S_{12} + S_{13} + S_{123} $$

Thus, this index adds to the first-order effect ${S}_{i}$ all interaction effects (i.e., higher-order sensitivity indices) that involve the considered input ${X}_{i}$. This implies that ${S}_{Ti}\ge {S}_{i}.$ If ${S}_{Ti}\approx 0$, it can be concluded that input factor $i$ has a negligible contribution to $V\left(Y\right)$ (Puy et al., 2021b, p. 2; Saltelli et al., 2008, p. 34). A notable difference between ${S}_{i}$ and ${S}_{Ti}$ indicates important interaction effects of the considered factor with one or several other factors. Investigating interaction effects enables a thorough analysis of the model structure (Saisana et al., 2005, p. 311). The higher the value of the sensitivity indices, ${S}_{i}$ and ${S}_{Ti}$, the more influential the respective input factor(s).

### Estimation of First- and Total-Order Sobol’ Indices

The search for first- and total-order estimators that are both efficient and robust is an active field of research (see Saltelli et al., 2010 and Prieur & Tarantola, 2015 for an overview). Azzini et al. (2020, p. 10) demonstrate the relevance of an estimator that allows only for values that are consistent with theory. Puy et al. (2021b) illustrate that the accuracy and efficiency of estimators can be influenced by several factors, such as the sampling method, the form and dimensionality of the model, the distribution and number of model inputs, as well as the number of model runs (p. 3f). They empirically compare eight MC-based estimators for the total-sensitivity index. The estimator developed by Azzini et al. (2020) is one of the best performing, both when the goal is to rank input factors according to their contribution to model output variance (factor prioritisation setting), as well as when the aim is to approximate the “true” indices (factor fixing setting) (p.12). Thus, the Azzini estimator is recommended by Puy et al. (2021b, p. 19), since it is both efficient and robust to a wide range of practical situations.

The Azzini estimators for the first- and total-order sensitivity indices can be summarized as follows.

The estimation procedure requires the creation of several sample matrices: ${\varvec{A}}, {\varvec{B}},\boldsymbol{ }{{\varvec{A}}}_{{\varvec{B}}}^{(i)} \,and \;{{\varvec{B}}}_{{\varvec{A}}}^{(i)}$, each of dimension $(N,k)$, where $N$ indicates the number of samples and $k$ denotes the number of input factors. These matrices are generated as follows. First, a $(N,2k)$ matrix of input factors is generated, where input factors are random and mutually independent. The first $k$ columns are alllocated to the ${\varvec{A}}$ matrix, and the remaining $k$ columns to matrix ${\varvec{B}}$. Then, $k$ additional matrices ${{\varvec{A}}}_{{\varvec{B}}}^{(i)}$ (${{\varvec{B}}}_{{\varvec{A}}}^{(i)}$) are created, where $k-1$ columns come from matrix ${\varvec{A}}$ (${\varvec{B}}$) and column $i$ comes from ${\varvec{B}}$ (${\varvec{A}}$). Hence, in each matrix, each row provides a set of scalar input factors ${\varvec{X}} = \left( {X_{1} , ...,X_{k} } \right)$ (i.e., a sample) that is used for one model run.

Furthermore, if we assume that we have one scalar output, denoted $Y = f\left( {\varvec{X}} \right)$, of a deterministic model then, the estimator for the first-order sensitivity index can be written as:

5 $$ \widehat{{S_{i} }} = \frac{{2\mathop \sum \nolimits_{\upsilon = 1}^{N} \left( {f\left( {{\varvec{B}}_{{\varvec{A}}}^{\left( i \right)} } \right)_{\upsilon } - f\left( {\varvec{B}} \right)_{\upsilon } } \right)^{2} + \left( {f\left( {\varvec{A}} \right)_{\upsilon } - f\left( {{\varvec{A}}_{{\varvec{B}}}^{\left( i \right)} } \right)_{\upsilon } } \right)^{2} }}{{\mathop \sum \nolimits_{\upsilon = 1}^{N} \left( {f\left( {\varvec{A}} \right)_{\upsilon } - f\left( {\varvec{B}} \right)_{\upsilon } } \right)^{2} + \left( {f\left( {{\varvec{B}}_{{\varvec{A}}}^{\left( i \right)} } \right)_{\upsilon } - f\left( {{\varvec{A}}_{{\varvec{B}}}^{\left( i \right)} } \right)_{\upsilon } } \right)^{2} }} $$

where, for instance, $f\left( {\varvec{A}} \right)_{\upsilon }$ denotes the model output $y$ obtained after running the model in the $\upsilon$-th row of the ${\varvec{A}}$ matrix. Furthermore, the total-order sensitivity index can be estimated as:

6 $$ \widehat{{S_{Ti} }} = \frac{{\mathop \sum \nolimits_{\upsilon = 1}^{N} \left( {f\left( {\varvec{B}} \right)_{\upsilon } - f\left( {{\varvec{B}}_{{\varvec{A}}}^{\left( i \right)} } \right)_{\upsilon } } \right)^{2} + \left( {f\left( {\varvec{A}} \right)_{\upsilon } - f\left( {{\varvec{A}}_{{\varvec{B}}}^{\left( i \right)} } \right)_{\upsilon } } \right)^{2} }}{{\mathop \sum \nolimits_{\upsilon = 1}^{N} \left( {f\left( {\varvec{A}} \right)_{\upsilon } - f\left( {\varvec{B}} \right)_{\upsilon } } \right)^{2} + \left( {f\left( {{\varvec{B}}_{{\varvec{A}}}^{\left( i \right)} } \right)_{\upsilon } - f\left( {{\varvec{A}}_{{\varvec{B}}}^{\left( i \right)} } \right)_{\upsilon } } \right)^{2} }} $$

Note that in both Eqs. 5 and 6, the denominator estimates the unconditional sample variance $\hat{V}\left( Y \right)$.

## Methodology

In the following section, we present the set-up of the Monte Carlo experiment, which provides the basis for the variance-based UA and SA. Its structure follows recommendations of the OECD *Handbook on constructing composite indicators* (2008). The procedure involves five steps.

### Step 1: Definition of the Model and Considered Model Outputs

In essence, index score $Y_{{\text{c}}}$ for country $c, c = 1,...M$ is calculated as a function of $Q$ normalised indicators $I_{{{\text{q}},{\text{c}}}}$ and weights $w_{{\text{q}}}$,

7 $$ Y_{{\text{c}}} = f\left( {I_{{{\text{q}},{\text{c}}}} ,w_{{\text{q}}} } \right) $$

In the case of the EQx, indicators are standardised through the application of z-scores, and then rescaled to fall within a $\left[ {0;100} \right]$ interval. A country’s score is then computed as the weighted arithmetic mean of all indicators. Note that in this representation, $w_{q}$ represents the final weight of an indicator within the index, as implied by the weights at each index level and the linear aggregation scheme. Thus, an indicator’s final weight is the result of multiplying the indicator-within-pillar weight with the pillar, index area and sub-index weight this indicator is associated with.^1^

Throughout the UA and SA, we investigate two model outputs: firstly, the rank assigned by the index to a given country, $rank\left( {Y_{c} } \right)$; and secondly, the average shift in country rankings, $\overline{R}$, calculated as the average of the absolute difference in countries' ranks with respect to the baseline EQx ranking, over all $M$ countries:

8 $$ \overline{R} = \frac{1}{M}\mathop \sum \limits_{{{\text{c}} = 1}}^{{\text{M}}} \left| {{\text{rank}}_{{{\text{EQx}}}} \left( {Y_{{\text{c}}} } \right) - {\text{rank}}\left( {Y_{{\text{c}}} } \right)} \right| $$

The above equations characterise the index, henceforth termed the model, and the investigation of $rank\left( {Y_{c} } \right)$ and $\overline{R}$ will be the scope of the UA and SA.

### Step 2: Definition of Input Factors

We consider the implications of uncertainty for the following model assumptions:

1. handling of missing values;
2. measurement error in the raw data;
3. omission of individual indicators;
4. choice of optima of selected indicators;
5. choice of normalisation scheme;
6. choice of weighting scheme;
7. choice of aggregation scheme.

These uncertainties are transferred into a set of $k$ input factors $X_{i} , i = 1,2, \ldots k$.

Input factor ${\text{X}}_{1}$ defines how missing values in the raw indicator data are handled. ${\text{X}}_{2}$ triggers whether random noise is added to the raw data (this approach is parallel to Saisana and Saltelli (2010, p. 3), and similar to Aguña & Kovacevic, 2011, p. 30).^2^${\text{X}}_{3}$ determines which indicator, if any, is omitted. The fourth input factor, ${\text{X}}_{4}$, selects one of three sets of optima applied to indicators with a conceptual optimum. Input factor ${\text{X}}_{5}$ triggers the normalisation scheme that is adopted, ${\text{X}}_{6}$ the weighting scheme, and ${\text{X}}_{7}$ selects the aggregation scheme at the sub-index level. Furthermore, we assign uniform distributions (discrete or continuous) to each input factor. Table 1 lists the input factors, along with their associated distribution and an explanation. Appendix 1 provides further detail on input factors ${\text{X}}_{1}$, $X_{4}$, $X_{5}$, $X_{6}$ and $X_{7}$.

**Table 1:** Summary of the uncertain input factors and their distributions

Input factor	Definition	Distribution
$X_{1}$	Imputation of missing values	${\mathcal{D}\mathcal{U}}\left[ {1,2} \right]$ where $1 \equiv$ handling of missing values according to EQx2021 methodology, $2 \equiv$ missing values fully imputed using predictive mean matching.
$X_{2}$	Measurement error	${\mathcal{D}\mathcal{U}}\left[ {1,2} \right]$ where $1 \equiv$ original raw data is used, $2 \equiv$ normally distributed random error with mean 0 and standard deviation equal to $1/5$ ^th^ of an indicator’s observed std. dev. is added to raw data.
$X_{3}$	Omission of individual indicators	${\mathcal{U}}\left[ {0,1} \right]$ where $[ {0,\frac{1}{{\left( {Q + 1} \right)}}} { } \equiv$ no indicator excluded, $[ {\frac{1}{{\left( {Q + 1} \right)}}, \frac{2}{{\left( {Q + 1} \right)}}} ){ } \equiv$ exclude indicator $I_{1}$, $( {...} )$ $[ {\frac{Q}{{\left( {Q + 1} \right)}}, 1} ]{ } \equiv$ exclude indicator $I_{Q}.$
$X_{4}$	Choice of conceptual optima	${\mathcal{D}\mathcal{U}}\left[ {1,2,3} \right]$ where $1 \equiv$ EQx conceptual optima where applicable, $2 \equiv$ EQx optima plus one std. dev. of the indicator’s raw data, $3 \equiv$ EQx optima minus one std. dev. of the indicator’s raw data.
$X_{5}$	Normalisation scheme	${\mathcal{D}\mathcal{U}}\left[ {1,2} \right]$ where (prior to rescaling values to range from 0 to 100) $1 \equiv$ EQx normalisation, $2 \equiv$ MinMax.
$X_{6}$	Weighting scheme	${\mathcal{D}\mathcal{U}}\left[ {1,2,...10} \right]$ where $1 \equiv$ EQx weighting, $2 \equiv$ equal indicator weights (Altern. 1), $3 \equiv$ equal pillar weights (Altern. 2), $4 \equiv$ equal indicator and pillar weights (Altern. 3), $5 \equiv$ equal indicator, pillar and index area weights (Altern. 4), $6 \equiv$ equal indicator, pillar, index area and sub-index weights (Altern. 5), $7 \equiv$ equal final indicator weights (Altern. 6), $8 \equiv$ Sub-index Power & Value at 0.25 & 0.75 (Altern. 7), $9 \equiv$ Sub-index Power & Value at 0.5 & 0.5 (Altern. 8), $10 \equiv$ equal index area and sub-index weights (Altern. 9).
$X_{7}$	Aggregation scheme	${\mathcal{D}\mathcal{U}}\left[ {1,2} \right]$ where $1 \equiv$ EQx aggregation (linear), $2 \equiv$ geometric aggregation at sub-index level.

${\mathcal{U}}$ stands for uniform, and ${\mathcal{D}\mathcal{U}}$ indicates a discrete uniform distribution

### Step 3: Generation of Independent Input Factors

For the implementation of the Monte Carlo experiment, combinations of input factors need to be sampled randomly and independently. This allows for the consideration of input factors as “stochastic variables so that the model induces a distribution in the output space” (Pianosi et al., 2016, p. 222), which is a basic principle of variance-based SA. Quasi-random sequences can be used in order to generate samples of the input factors as uniformly as possible over the unit hypercube ${\Omega }$ (Saltelli et al., 2010, p. 263). Saltelli et al. (2010, p. 263) propose using Sobol’s quasi-random sequence, following Sobol’ & Kucherenko (2005). Using the Sobol’ sampling scheme yields “faster convergence and better accuracy” when conducting a SA (Zhang et al., 2015, p. 72) and can be considered best practice (Puy et al., 2021b, p. 11).

Hence, the sample matrices required to apply the Azzini estimators are generated using Sobol’ quasi-random numbers. Then, each input factor is transformed to its specific probability distribution. As a result, in the sample matrices, each column is a model input factor described with the probability distributions of Table 1 and each row gives one sample that is used for one iteration of the experiment.

The total number of simulations conducted in the Monte Carlo experiment depends on $N$ (the number of samples) and $k$ (the number of model inputs), and amounts to $2N\left( {k + 1} \right)$ when applying the Azzini estimator. Regarding the choice of $N$, Zhang et al. (2015, p. 74) note that: “there is no general consensus on the optimal number of parameter sets to be generated, the general rule of thumb is that the larger numbers of model parameters, the higher the number of parameter sets to be used”. Since we will bootstrap the Sobol’ indices to obtain confidence intervals, we set the sample size relatively high, to $N = 5^{\prime}000$, which implies a total of $80^{\prime}000$ model runs in the course of the Monte Carlo experiment.

### Step 4: Evaluation of the Model

In this step, the model is repeatedly evaluated based on the generated input factor samples. We consider two model outputs: $rank\left( {CI_{c} } \right)$ and $\overline{R}_{S}$ (see Step 1). Hereinafter, we denote the index computed using any realisation of uncertain input factors as Monte Carlo EQx (MCEQx).

### Step 5: Analysis of the Output

The previous step results in an output vector ${\varvec{Y}}^{l}$ that builds an empirical pdf of output $Y$ that can be analysed. A descriptive analysis of its characteristics provides the basis of the uncertainty analysis, quantifying the uncertainty in the model output. The analysis of higher orders of the pdf of output $Y$ constitutes the sensitivity analysis, apportioning the uncertainty in the model output to the different input factors. Performing a variance-based SA, the first-order sensitivity index $S_{i}$ as well as the total effect sensitivity index $S_{Ti}$ will be computed and analysed. We estimate $S_{i}$ and $S_{Ti}$ based on the formulas presented by Azzini et al. (2020)^3^

## Results

Index scores and ranks are computed for all 151 countries considered by the EQx2021, for each of the 80′000 random combinations of input factors, resulting in the MCEQx. While the MCEQx relies on the EQx theoretical framework as well as the overall choice of indicators, it represents a large range of plausible methodological choices. Hence, this allows us to compare the EQx as implied by the EQx methodology, to index results largely independent of methodology. The next section (UA) aims to quantify and illustrate the overall uncertainty in the index ranking as a result of the uncertainty in the methodological choices. In a second step, the SA apportions this uncertainty to individual or the interaction of methodological choices in the index set-up.

### Uncertainty Analysis

**Fig. 1:**
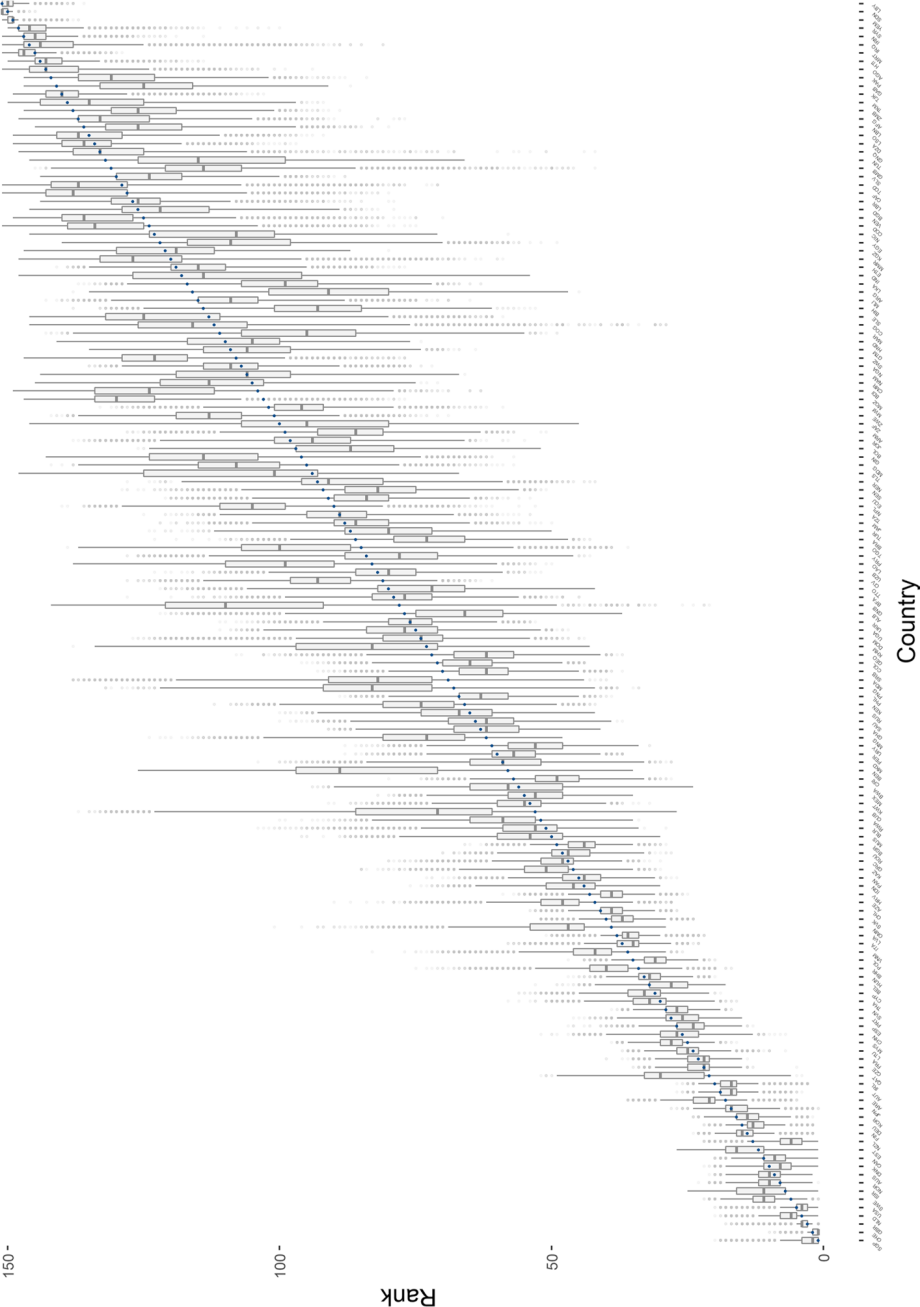
Boxplot of MCEQx ranks per country. Note: Countries are ordered by their EQx2021 rank, indicated by the blue dot. Grey boxplots illustrate the distribution of the MCEQx: boxes include 50% of a country’s MCEQx ranks, and the horizontal line within a box indicates the a country’s median MCEQx rank. Whiskers span up to ± 1.5 times the interquartile range (IQR: Q3–Q1), up to the lower observed point from the MCEQx ranks that falls within this distance. All other observed ranks are plotted as outliers (grey dots)

The results of the uncertainty analysis from the Monte Carlo simulations are illustrated in Fig. 1, which compares the EQx2021 ranking with the distribution of ranks implied by the MCEQx. Figure 1 shows that countries ranking in the top 50 of the EQx2021 have a comparatively low variation in MCEQx ranks. Grey boxes, denoting 50% of MCEQx ranks, are relatively small and, in most cases, overlapp or remain fairly close to the EQx2021 rank. However, starting with Cuba, the variation in MCEQx ranks appears to be alarmingly high.

Furthermore, a substantial number of countries have their original rank placed outside the grey box, sometimes by a sizeable distance, implying that in these cases, the country was ranked similarly to their original position in less than half of all simulations. Interestingly, countries in the bottom 10 of the EQx2021 ranking again fluctuate much less when ranked using alternative methodological choices.

Appendix A2 allows to inspect the uncertainty in the EQx2021 ranking in more detail. For each country, the table shows the frequency of it being among the top 10, top 11–20, and so on, in the MCEQx ranking. For instance, Singapore, ranked 1st in the EQx2021, is among the top 10 performing countries in 92.4% of the Monte Carlo simulations. Generally, the EQx2021 top 10 appears rather robust, since, with the exception of Sweden and Israel, all EQx2021 top 10 countries are also ranked among the top 10 in more than half of all of the simulations. Moreover, the ranking of the top 50 countries seems largely robust: 35 out of 50 countries are placed correctly in more than 50% of the simulations. However, as already illustrated in Fig. 1, this overall stability in the ranks is dramatically reduced for countries below rank 50, except for countries in the bottom 10: of all countries ranked 51 to 140 in the EQx2021, only eight are placed correctly in more than 50% of the simulations.

This invites us to have a closer look at the ‘winners’ and ‘losers’ of the EQx methodology, that is, countries whose EQx2021 rank lies higher or lower than the one largely implied by the MCEQx. Table 2 lists countries whose EQx rank lies outside the range of 1 standard deviation around the country’s MCEQx median rank. Countries are ordered according the distance between their EQx rank, and the range largely implied by the MCEQx. Mozambique, Benin, and Guinea-Bissau are, by far, the biggest ‘winners’, since their EQx2021 rank is between 14 and 17 places higher than the upper end of the 1 standard deviation range around the MCEQx median. Guinea-Bissau and Eswatini benefit to a lesser extent, with an EQx2021 rank of 5 to 6 places better than implied by the MCEQx. On the other hand, Argentina, Bosnia and Herzegovina, Sri Lanka, Gabon, and Gambia have an EQx2021 rank between 10 and 5 places lower than suggested by the MCEQx. These countries’ ranks appear to be ‘dragged down’ by the EQx methodology.

**Table 2:** ‘Winners’ and ‘losers’ of the EQx methodology

Countries ‘favoured’			Countries ‘disadvantaged’		
Country	EQx rank	Range of 1 std.dev. around the median MCEQx rank	Country	EQx rank	Range of 1 std.dev. around the median MCEQx rank
Mozambique	103	[119.92, 140.08]	Argentina	116	[76.27, 105.73]
Benin	58	[72.94, 105.06]	Bosnia and Herzegovina	114	[81.44, 104.56]
Guinea-Bissau	78	[92.34, 127.66]	Sri Lanka	117	[89.01, 108.99]
Eswatini	108	[113.77, 132.23]	Gabon	141	[115.13, 134.87]
Burundi	104	[109.24, 138.76]	Gambia, The	131	[102.47, 125.53]
Nepal	90	[94.3, 115.7]	Brazil	86	[63.7, 82.3]
Côte d'Ivoire	81	[85.29, 100.71]	New Zealand	13	[2.38, 9.62]
Zimbabwe	101	[104.27, 121.73]	Zambia	138	[117.09, 134.91]
Guinea	96	[99.19, 128.81]	Armenia	99	[75.63, 96.37]
Lao PDR	83	[85.58, 112.42]	Costa Rica	57	[43.34, 54.66]
Qatar	21	[23.18, 36.82]	Pakistan	142	[121.65, 140.35]
Sweden	6	[7.76, 14.24]	Georgia	72	[53.34, 70.66]
Madagascar	95	[96.64, 119.36]	Poland	35	[28.28, 33.72]
Papua New Guinea	68	[69.44, 96.56]	Bulgaria	49	[40.2, 47.8]
Mongolia	62	[62.84, 83.16]	Uruguay	61	[46.06, 59.94]
Venezuela, RB	125	[125.79, 146.21]	Tunisia	132	[98.98, 131.02]
Sudan	150	[150.29, 151.71]	Nicaragua	123	[93.87, 122.13]
Oman	39	[39.26, 54.74]	Lebanon	136	[116.82, 135.18]
Vietnam	36	[36.24, 47.76]	Croatia	43	[35.74, 42.26]
Malaysia	25	[25.22, 30.78]	Serbia	70	[54.73, 69.27]
			Switzerland	2	[0.32, 1.68]
			Morocco	111	[79.32, 110.68]
			Senegal	92	[72.24, 91.76]

Table shows countries whose EQx2021 rank lies outside the range of the median MCEQx, ± 1 standard deviation (std. dev.)

Furthermore, Fig. 1 illustrates that the range of ranks that a country can hold is substantially larger for countries in the middle and lower performing sections of the EQx2021 ranking. This leads us to investigate a country's volatility in the ranking, by measuring the difference between a country's best and worst rank, calculated from the 5th and 95th percentiles in the MCEQx rank distribution.

Table 3 lists the 20 countries most affected by the EQx2021 methodological choices, by highlighting those with the widest distance between their 5th and 95th percentile ranks in the MCEQx rank distribution. The ranks of Cuba, India and Cambodia are most affected by the methodological set-up. Cuba, for instance, has an EQx2021 rank at the upper end of the MCEQx ranking distribution (53^rd^ rank), but ranks between 50 and 111 in the MCEQx. All of the countries listed in Table 3 are middle and low-performing countries in the EQx2021, from Cuba (rank 53) to Tunisia (rank 132).

**Table 3:** Top 20 countries most affected by methodology choices

Country	EQx rank	MCEQx 5th and 95th percentile ranks
Cuba	53	[50, 111]
India	118	[76, 136]
Cambodia	73	[57, 114]
Moldova	69	[60, 116]
South Africa	100	[66, 122]
Timor-Leste	94	[87, 142]
Egypt, Arab Rep	122	[75, 129]
Morocco	111	[69, 123]
Togo	85	[62, 116]
Sierra Leone	113	[84, 137]
Congo, Rep	112	[82, 134]
Guinea-Bissau	78	[77, 129]
Benin	58	[56, 105]
Tunisia	132	[86, 135]
Albania	77	[51, 99]
Argentina	116	[68, 116]
Burundi	104	[94, 142]
Guinea	96	[82, 130]
Paraguay	84	[62, 109]
Lao PDR	83	[78, 122]

Table lists the 20 countries with the largest difference between the 5th and 95th percentile ranks of the MCEQx ranking distribution. Countries are ordered according to their range of MCEQx ranks

Furthermore, we aim to provide guidance regarding the reliability of the EQx2021 ranking and how it is interpreted. Thus, Table 7 in the Appendix presents the EQx2021 ranks after accounting for uncertainty considerations, with countries colour-coded according to the robustness of their rank. In short, countries scoring particularly highly (in the top 50) in the EQx2021 have a relatively small range of ranks under alternative methodological choices. However, countries especially in the middle and lower performing sections of the EQx2021 ranking exhibit a large range of possible ranks depending on the methodological set-up, implying that any interpretation or conclusion on the elite quality of these countries should be treated with caution.

### Sensitivity Analysis

The UA has revealed considerable uncertainty in the EQx2021 ranking due to uncertainties in the methodology. This section presents a variance-based SA in order to identify the input factors that contribute most to that variation of the model output $\overline{R}$, the average shift in country ranks.

Table 4 shows the Sobol’ sensitivity indices of first-order effect ($$S_{i}$$) and total effect ($$S_{iT}$$) for the average shift in country ranks ($$\overline{R}$$). Note that to distinguish relevant input factors from less important ones, Sobol’ sensitivity indices do not rely on an absolute threshold, but rather indicate the relative importance of input factors. The first-order effect indicates the share by which output variance could be reduced if the considered input factor could be fixed individually. None of the first-order sensitivity indices is equal to 1, which implies that no input factor uniquely determines the index ranking. Taken individually, the input factors determining the normalisation ($$X_{5} )$$ and the weighting scheme ($$X_{6}$$) are by far the most important, explaining 19.6% and 40% of output variance respectively. All input factors, taken individually, account for 70.3% of output variance. Since this indicates index calculation to be a non-additive model, this also confirms the relevance of applying a global UA and SA. The remaining share of output variance of 29.7% is explained by interactions between input factors.

**Table 4:** Sobol' sensitivity indices for output $\overline{R}$ (average shift in country ranks)

Input factor	Definition	First-order effect $S_{i}$		Total effect $S_{Ti}$		$S_{Ti} - S_{i}$
$X_{1}$	Imputation of missing values	0.038	(0.006)	0.301	(0.007)	0.264
$X_{2}$	Measurement error	0.045	(0.002)	0.082	(0.002)	0.036
$X_{3}$	Omission of individual indicators	0.007	(0.001)	0.029	(0.001)	0.023
$X_{4}$	Choice of conceptual optima	0.016	(0.001)	0.044	(0.001)	0.028
$X_{5}$	Normalisation scheme	0.196	(0.008)	0.465	(0.01)	0.269
$X_{6}$	Weighting scheme	0.400	(0.007)	0.476	(0.007)	0.076
$X_{7}$	Aggregation scheme	0.002	(0.006)	0.018	(0.007)	0.016
Sum		0.703		1.416		0.712

Table lists sensitivity indices, their standard errors (in brackets), and the difference between first- and total order indices. Numbers are rounded to three decimals

Thus, we inspect $S_{iT}$, which adds to an input factor’s $S_{i}$ all interaction effects that involve said input factor. The total effect sensitivity index is close to zero for the input factors determining the potential measurement error ($X_{2}$), the omission of indicators ($X_{3}$), the choice of conceptual optima ($X_{4}$) and the aggregation scheme at the sub-index level ($X_{7}$). Hence, the influence of these methodological choices is relatively negligible, and these input factors can be declared non-influential (Pianosi et al., 2016, p. 222). Accounting for interaction effects, the most influential input factors are those that trigger the imputation of missing values ($X_{1}$), the normalisation ($X_{5}$) and the weighting scheme ($X_{6}$). A notable difference between $S_{i}$ and $S_{iT}$ indicates interaction effects with one or several other inputs. Interestingly, $X_{6}$ contributes to the output variance mainly individually, not via interaction effects, while conversely the importance of $X_{5}$ is largely due to interaction effects. Remarkably, $X_{1}$ contributes to output variance solely by interacting with the weighting and especially the normalisation scheme.

**Fig. 2:**
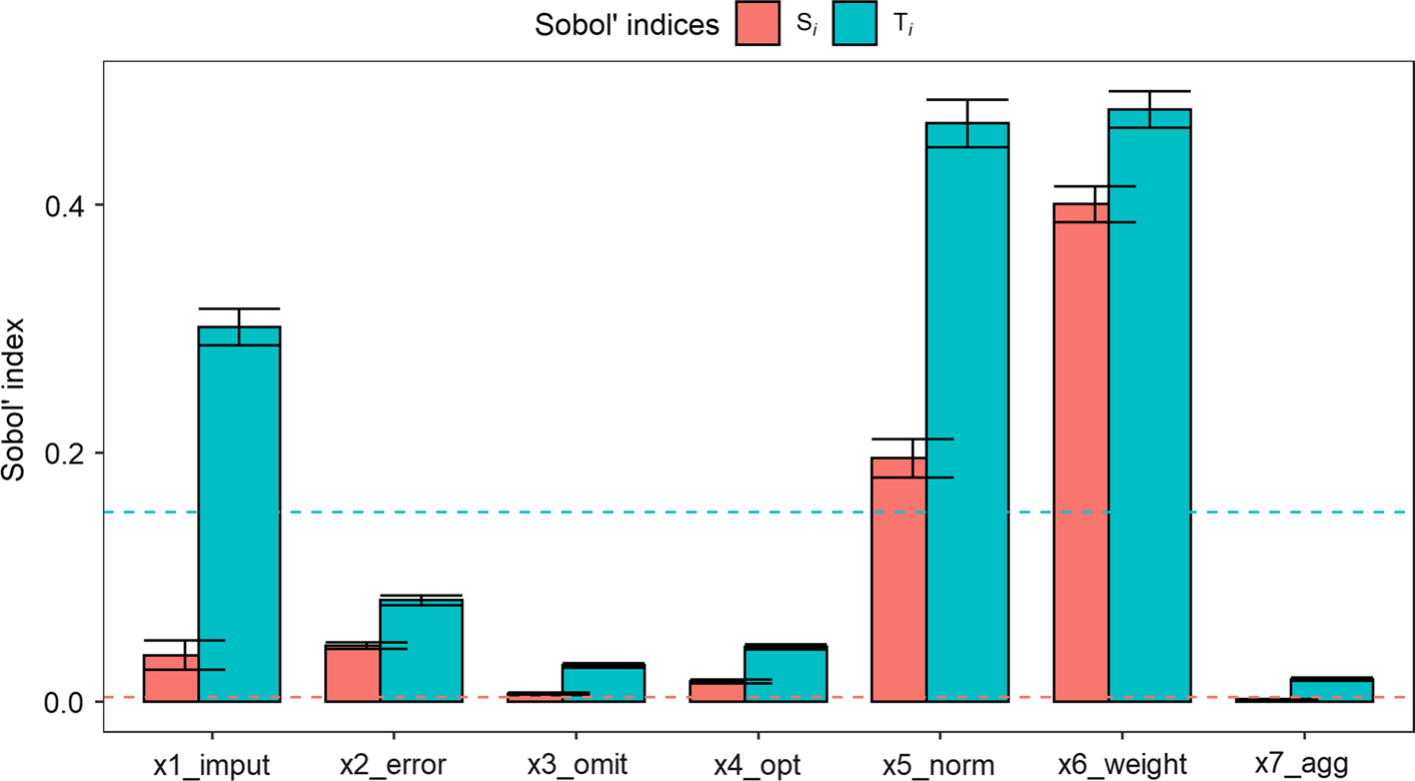
Sobol' sensitivity indices

The above findings are illustrated in Fig. 2, which plots the Sobol’ sensitivity indices for each input factor as well as their confidence intervals obtained from the bootstrap technique. In order to estimate the numerical approximation error, we additionally compute Sobol’ sensitivity indices for a dummy input factor that has no influence on the index ranking. The estimate of the dummy input factor is visualised through the use of a dashed line. This allows us to identify and visualise those input factors whose contribution to the output variance is less than the approximation error. Figure 2 highlights that whether or not missing values are imputed contributes to output variance solely by interacting with one or several other input factors. The normalisation and weighting scheme are the most important methodological choices, with the impact of the latter, considered singly, is even larger than the total effect of the imputation scheme.

### Discussion of the Results

What do our results imply for the EQx methodology? Generally, it must be mentioned that judgement calls are inevitable throughout any index construction process. Thus, it is often difficult to argue why one scheme is chosen over another, and, in consequence, “transparency must be the guiding principle of the entire exercise” (OECD, 2008, p. 17).

Among the three most important input factors, the weighting scheme can arguably be most convincingly rooted in the theoretical framework. Indeed, in the case of the EQx, the weights, particularly at the index area and sub-index levels, are deduced from conceptual deliberations (Casas et al., 2020). On the other hand, it may be more difficult to legitimise applying one particular approach to missing values or a normalisation method, over another, with convincing arguments. Since a large amount of uncertainty stems from the interaction between how missing values are handled and the normalisation scheme, a promising approach in improving the meaningfulness and robustness of the EQx ranking is to reduce the index’ sensitivity towards how missing values are addressed. This can most intuitively be achieved by reducing the amount of missing values in the EQx indicator data, which currently amounts to 26% of the overall indicator dataset.

There are further reasons to favour this approach. Generally, indicators that cover a set of heterogenous countries imply that each index score relies on a different set of indicators (Little & Rubin, 2002, p. 54). This might hamper the cross-country comparability of index scores. To address this concern, the EQx approach to missing values relies on the premise that indicators within a pillar measure a roughly similar aspect of elite quality. In consequence, missing values would “just” (Casas & Cozzi, 2021, p. 21) increase the noise rather than the bias of scores. However, our results suggest that, at least for a number of middle and lower performing countries in the EQx2021, the scores might actually be noised to such an extent that a reliable interpretation of the ranking might not be possible. In this respect it should be noted that index scores *per se* are seldom meaningful, but rather reveal information on the performance of a country in relation to other countries’ scores. Additionally, data availability often varies not only in terms of country-coverage, but also fluctuates from year to year. Hence, any attempt to reduce the number of missing values will not only contribute to the reliability of the ranking in a given year, but also render an analysis of elite quality over time more precise.

### Convergence and robustness of Estimated Sensitivity Indices

**Fig. 3:**
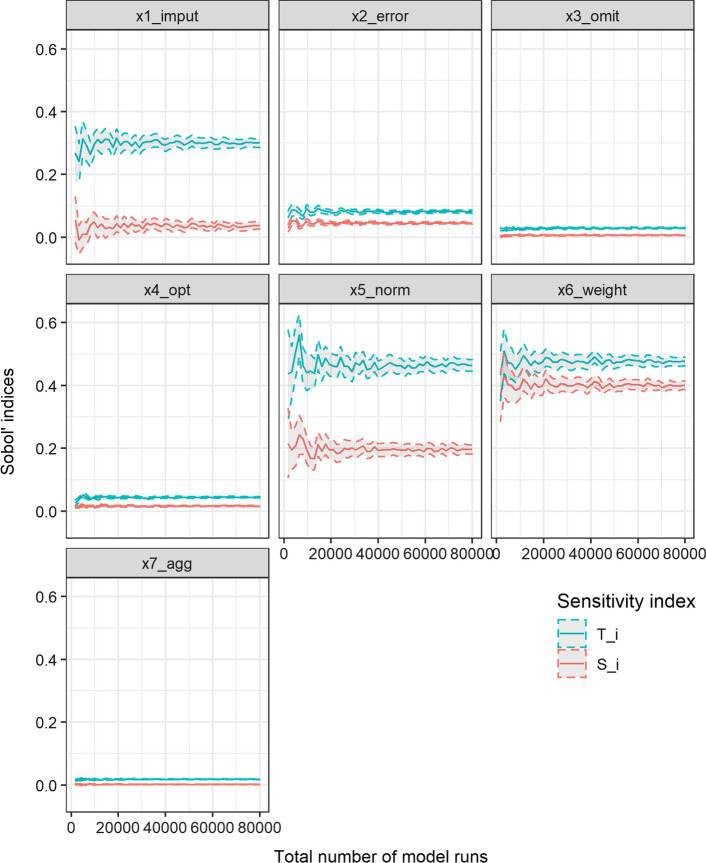
Robustness and convergence of estimated Sobol' sensitivity indices

When conducting a variance-based SA through Monte Carlo simulations, it is essential to check the convergence and robustness of the estimated sensitivity indices (Pianosi et al., 2016, p. 226; Sarrazin et al., 2016; Yang, 2011). Analysis of convergences ensures that estimated indices are independent of the underlying sample size, while robustness analysis ensures that the estimated indices are independent of the specific sample (Pianosi et al., 2016, p. 226). We apply the bootstrap technique presented in Yang (2011). Thus, Fig. 3 plots the estimated Sobol’ sensitivity indices resulting from gradually increasing sub-samples extracted from the original sample. Convergence can be assumed once the estimates stabilise and there is no serious variation (Pianosi et al., 2016, p. 226; Yang, 2011, p. 448). However, it could be that we obtain similar bootstrap means “‘by chance’ while the actual statistical convergence is not reached yet” (Sarrazin et al., 2016, p. 144). Thus, in order to analyse the robustness of the estimated indices, Fig. 3 additionally displays the indices’ 95% confidence intervals, constructed from the bootstrap estimate of the sampling distribution of the indices.

Since, for all input factors and the full sample size, the plotted estimates are stable and the confidence bounds narrow, we can confirm the convergence and robustness of the estimated Sobol’ sensitivity indices.

## Conclusion

The EQx2021 has attracted much media and press attention worldwide. We have conducted a global uncertainty and sensitivity analysis in order to examine how reliable and meaningful the ranking of the 151 analysed countries is that media coverage has mostly focused on. Specifically, we conducted a Monte Carlo experiment where we computed the index 80′000 times based on a randomly selected methodological set-up. We considered a range of plausible alternatives to the EQx methodology, and investigated their individual and combined effects on the country ranking.

The UA revealed that the ranking for the top 50 countries in the EQx2021 is largely robust to changes in key modelling assumptions. A more differentiated picture was found for countries in the middle and lower performing sections of the EQx2021. Their ranks are more sensitive to methodological choices, with alarming outliers in some cases. Any interpretation or conclusion on the elite quality of these particular countries should therefore be treated with caution.

In order to identify the input factors that exert the largest influence on the ranking, we conducted a variance-based SA. We estimated Sobol’ first- and total effect sensitivity indices using the state of the art Azzini et al. (2020) estimator. The SA revealed that a potential measurement error, the omission of individual indicators, different conceptual optima used for selected EQx indicators, as well as the aggregation method at the sub-index level have negligible effects on the country ranking. On the other hand, the handling of missing values, the normalisation scheme applied to raw indicators, and the weighting scheme have a substantially greater effect and are the most important choices in the EQx methodology. Remarkably, the handling of missing values contributes to output variance solely by interacting with the weighting and especially the normalisation scheme. Further analysis suggests that the estimated Sobol’ sensitivity indices converge and are robust.

Since it is often difficult to justify using one weighting or normalisation scheme over another in how an index is constructed, we argue that the most promising approach for improving the meaningfulness and robustness of the EQx is to reduce the index’ sensitivity towards the handling of missing values. This can be achieved either by improving the data coverage of existing indicators or, alternatively, reducing the indicator list to reflect a more narrow but, as a result, a more concise picture of elite quality. This would not only increase the explanatory power of the EQx ranking, but also allow for more meaningful evaluations and comparisons of elite quality over time.

This paper has set out to discuss the statistical properties of the methodological set-up of the EQx. There are several limitations to our results.

First, while the UA and SA investigate the implications of random measurement errors in the original indicator data, the analyses do not account for possible systematic biases present in the data that can potentially distort the country ranking. Constant efforts to ensure the use of the highest possible quality data are vital for the meaningfulness of all composite indicators, including the EQx.

Second, this study has analysed the meaningfulness of the EQx2021 ranking and has made concrete suggestions for a gradual improvement of its set-up. Yet, further development on its methodology might aim at endogenising the uncertainty in index assumptions. For instance, one could construct an interval-based composite indicator following the examples set by Drago (2021) and Gatto & Drago (2020), where the former attempts to measure the demand for support measures in response to the Covid-19 crisis in Italy, and the latter, energy resilience. These works compute a multitude of country scores implied by different weighting schemes, yielding an interval of possible index scores for each country. Thus, they present three country rankings: one based on a country’s minimum score; one on its maximum; and a third based on the average of the minimum and maximum scores. They argue that basing index values on an interval of scores instead of single values allows for country rankings to reflect the underlying uncertainties.

Third, this study has not challenged the underlying theoretical framework behind the EQx (Casas, forthcoming 2022) which defines the concept of elite quality. More specifically, the multi-layered index architecture implies country rankings at the sub-index, index area, pillar and indicator level, whereas this analysis has only evaluated the overall EQx2021 country ranking. An assessment of these more granular aspects is, however, beyond the scope of this study and is left for future research.

## Figures and Tables

**Table 7:**
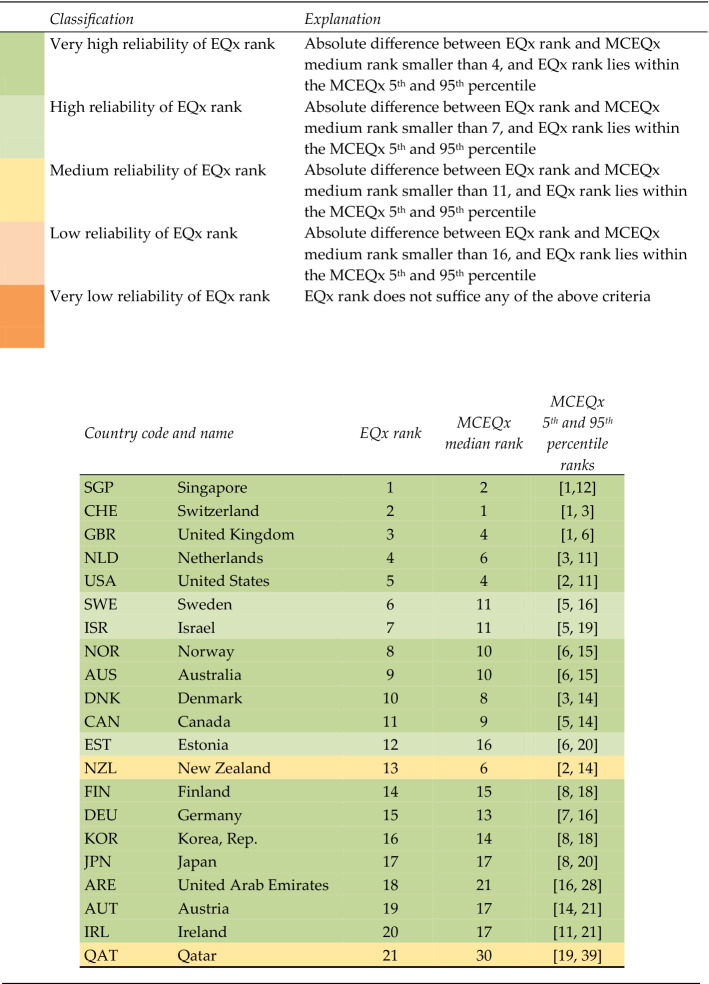

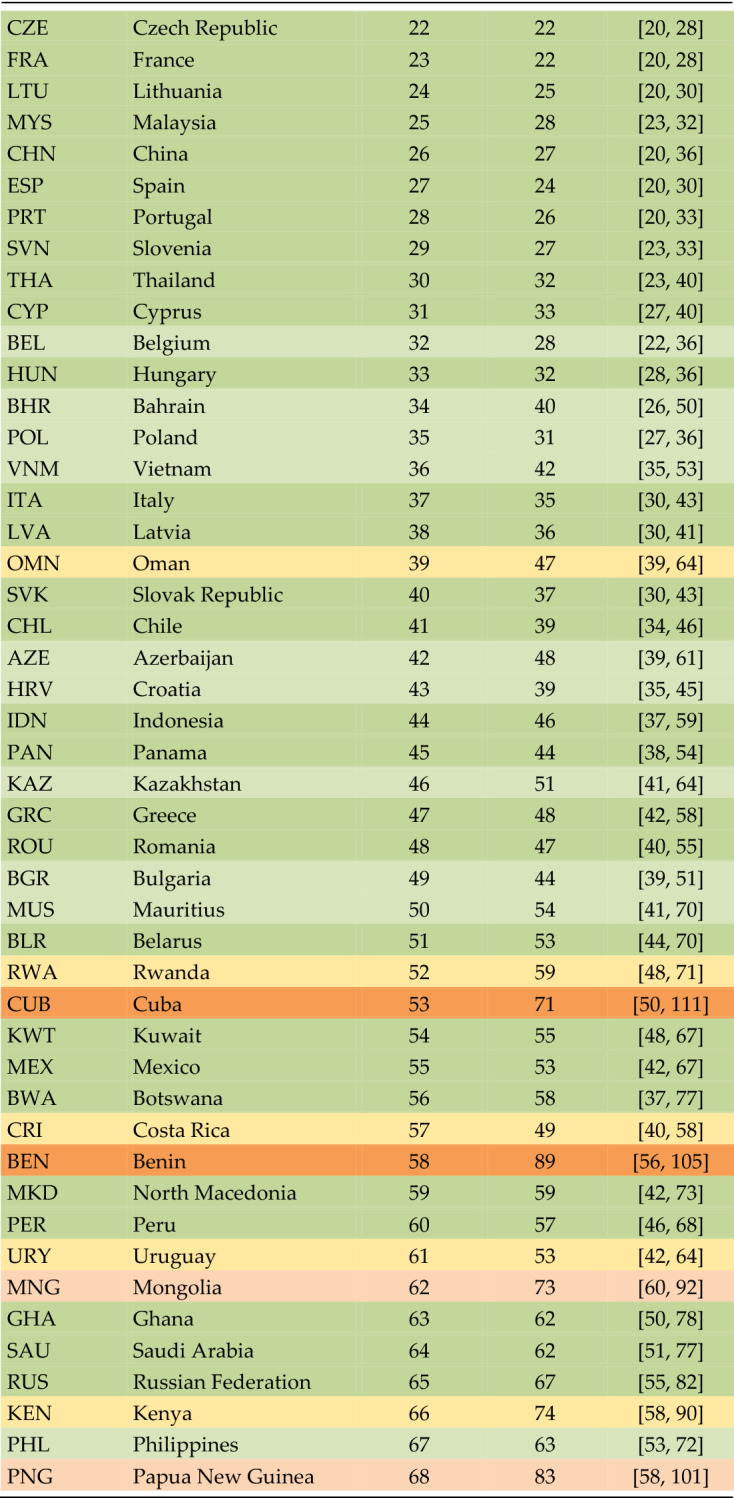

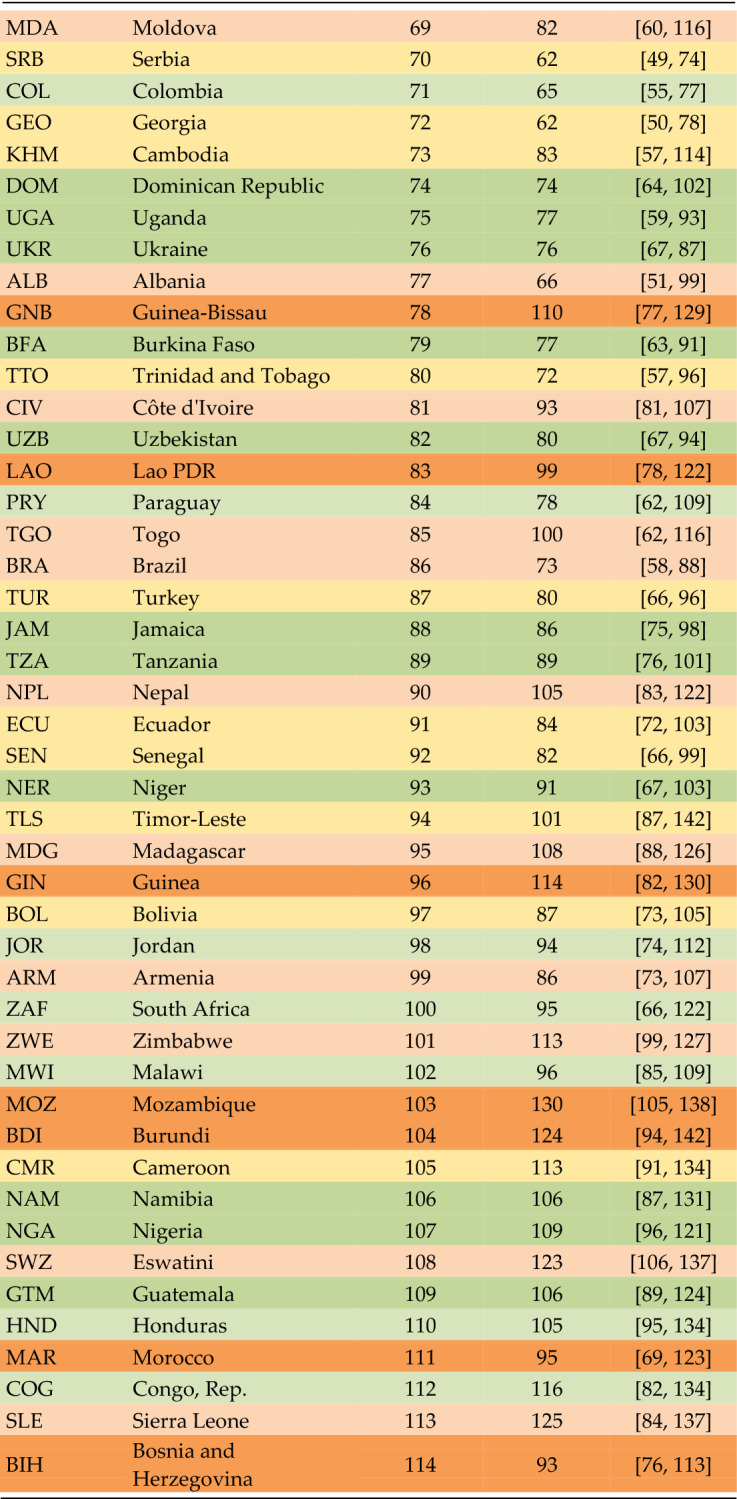

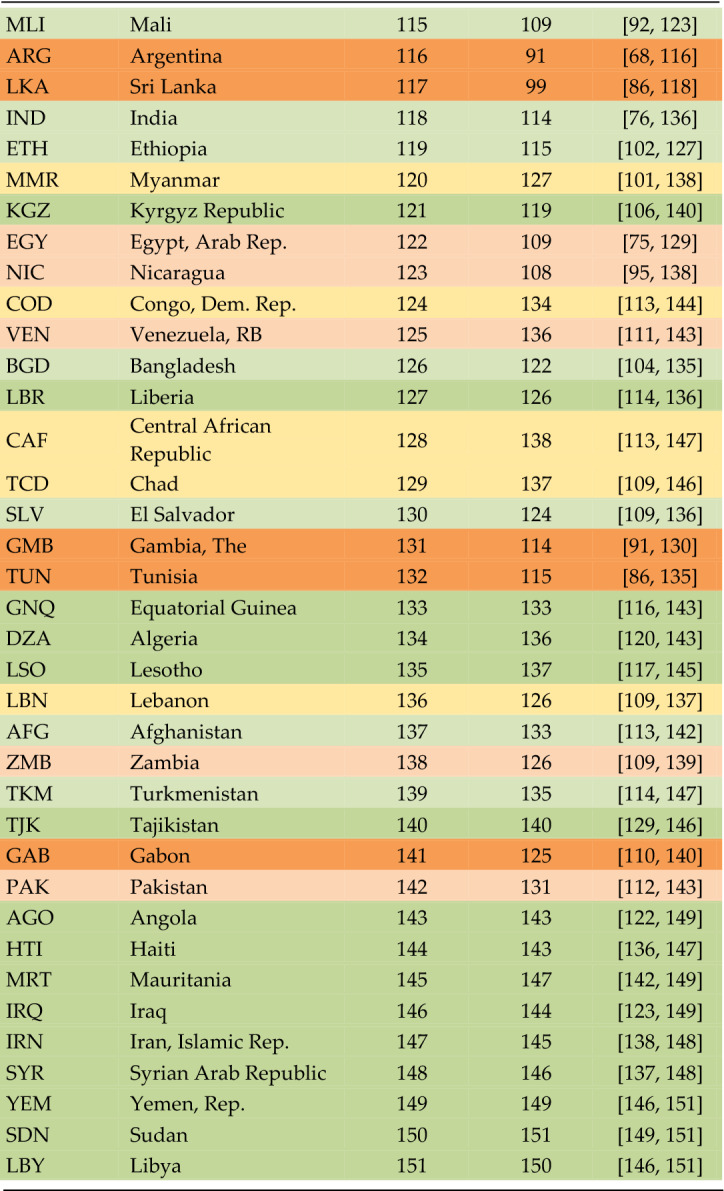
EQx2021 ranking, accounting for plausible range of methodological choices

Note: Table lists each country’s EQx rank, MCEQx median rank and 5th and 95th percentile ranks of a country’s MCEQx rank distribution. Countries are coloured according to the robustness of their rank. The less a country’s rank is reliable, that is, the more a country’s rank is affected by the underlying methodological choices, the more it should be treated with caution

**Fig. 4:**
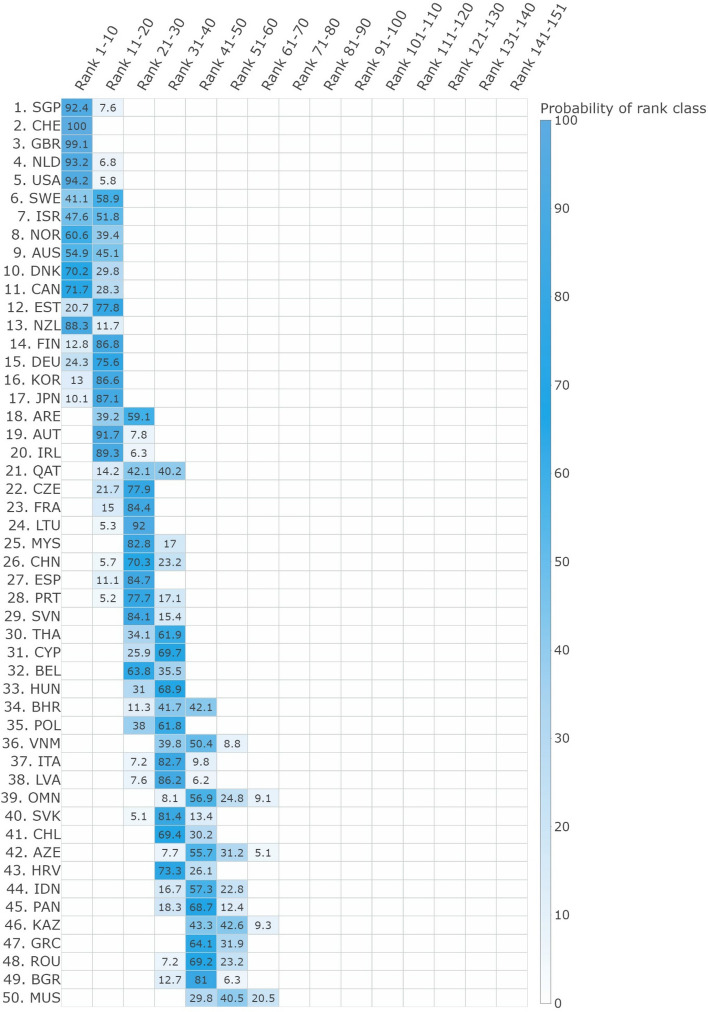

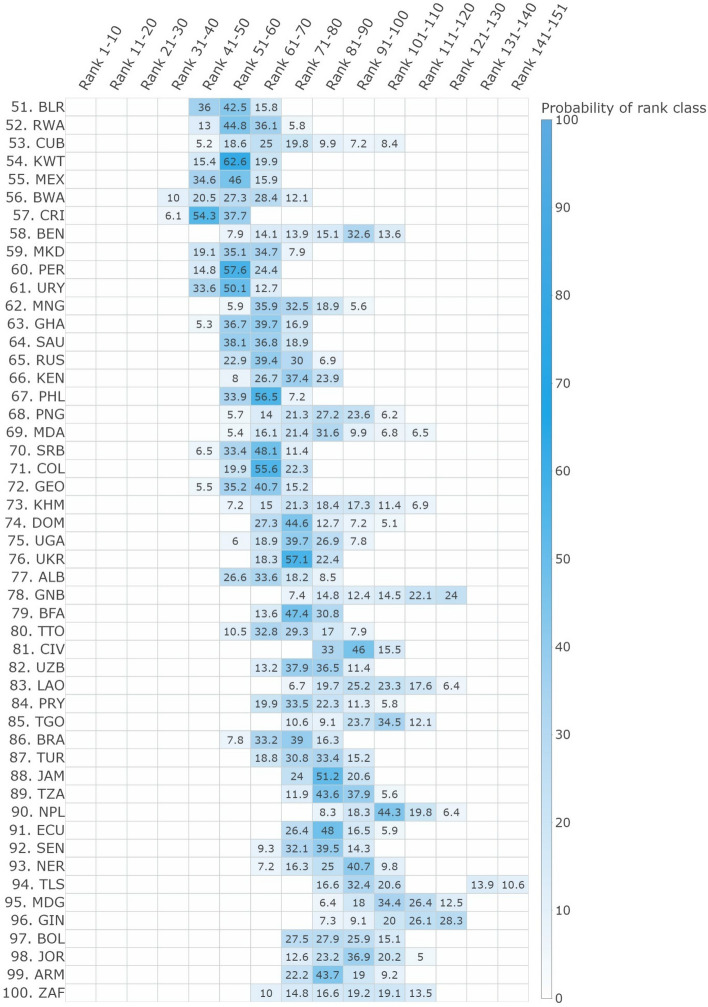

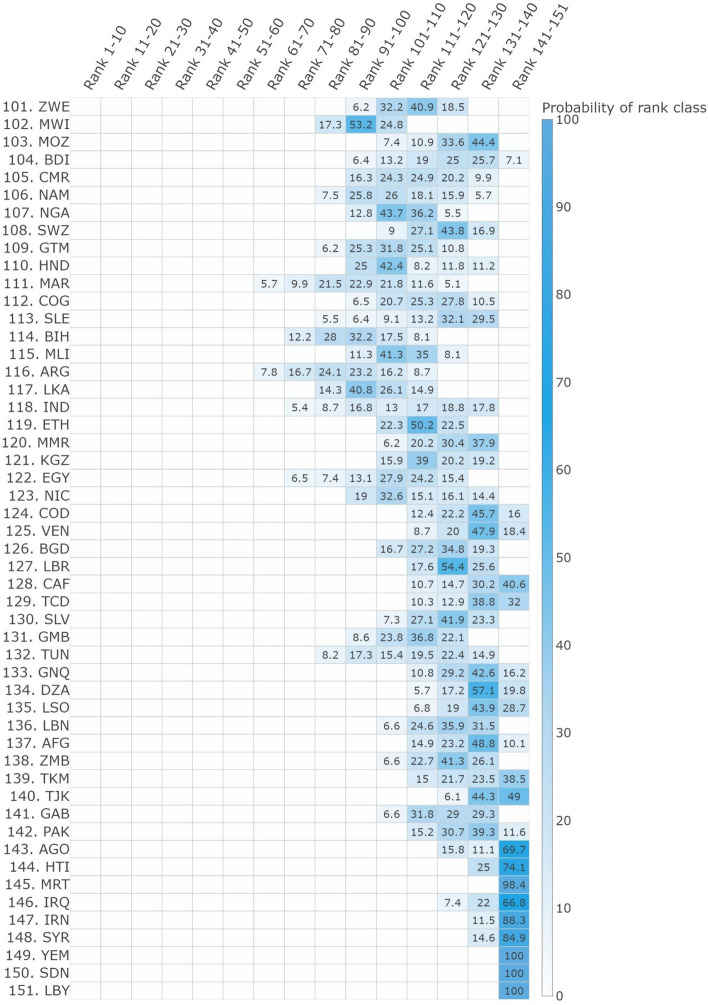
Frequency of MCEQx ranks per country

## Data Availability

In the process of replicating the computation of the Elite Quality Index, only publicly available data was used.

## A1 Explanation of Input Factors

### ${\varvec{X}}_{1}$—Imputation of Missing Values

Careful consideration needs to be given to the handling of missing values. The EQx2021 approach to missing data encompasses three aspects. Firstly, minimum data requirements are defined: Datasets are only considered if they cover a minimum of 15% of the countries under consideration, and provide recent information on a countries’ elite quality, i.e., no later than 2017 (although there are some exceptions to this rule). Furthermore, countries are only included if their index score is based on at least 40 datapoints, and more specifically, at least 3 datapoints per index area and 1 datapoint per pillar in at least 11 pillars. Secondly, if recent data is unavailable for only a small number of countries, missing datapoints are imputed with the latest available data, up to 3 years prior to the most recent year. Thirdly, the EQx2021 implements an “available-case analysis” (Little & Rubin, 2002, p. 54), where indicators are not omitted if they have missing values, but used if they fulfil the above minimum requirements. As a consequence, if the value of an indicator is missing for a particular country, the weight of the missing indicator is distributed among the remaining indicators of the same pillar, in proportion to their respective weights. The EQx methodology thus builds on the premise that indicators within the same pillar measure similar aspects of elite quality. For the EQx2021, a country’s score is derived from a minimum of 41 datapoints (the case of Turkmenistan), with an overall average of 79.2 datapoints for all countries considered.

However, the first two steps outlined above still leave the missing rate in the indicator dataset at roughly 26%. Hence, input factor $X_{1}$ triggers whether or not an alternative approach to handling these remaining missing indicator values is employed. Instead of allocating the weight of a missing indicator to the remaining existing indicators of the same pillar, missing indicator values are explicitly imputed, based on the existing, raw data. To impute missing values for composite indices, one approach recommended by the OECD (2008, p. 60ff) is multiple imputation. This method imputes missing data based on a random process, which reflects the uncertainty of missing data. The imputation algorithm is repeated independently multiple times, creating $M$ data sets where all missing values have been imputed. Finally, the $M$ imputed data sets are combined by taking the average, yielding one new set of complete data. More specifically, we employ an iterative *Markov Chain Monte Carlo* (MCMC) imputation algorithm (see van Buuren, 2018, p. 149ff, for the algorithm applied).

We use predictive mean matching (PMM) as the underlying imputation method. This nonparametric technique has several advantages: it is particularly suitable to impute normally distributed variables, but also if the underlying original data is somewhat skewed, which is sometimes the case for raw indicator values. PMM preserves the original distribution of the data. Furthermore, it respects potential bounds (if the original data, for instance, only contains non-negative values, only non-negative values are imputed) as well as whether initial values are discrete, continuous or semi-continuous (Vink et al., 2014). This is because PMM draws or “borrows” (Morris et al., 2014, p. 1) an imputed value from an original observation with a similar predictive mean. We follow the recommendations of Morris et al. (2014) by sampling an imputation from a pool of 10 donors.

### ${\varvec{X}}_{4}$—Choice of Conceptual Optima

Most EQx2021 indicators measure value creation or extraction linearly, so that, prior to normalisation, they can be typified as ‘the higher, the better’ or ‘the lower, the better’. Since this does not do justice to the ambivalent nature of several indicators, eight EQx indicators are defined as a ‘deviation from optimum’, where the lower the difference between a country’s indicator value and the conceptual optimum, the better. Ideally, this facilitates the capture of the detrimental effects of values being either higher or lower than some reference level. Table 5 lists the baseline EQx2021 set of optima, as well as the standard deviation of the underlying raw indicator data. The two alternative set of optima considered for the MCEQx are computed by adding or subtracting one standard deviation of the respective raw indicator data to the baseline optima.

**Table 5:** EQx2021 conceptual optima and indicator standard deviation

Indicator code	Indicator name	EQx2021 Optimum	Std. Dev
ii.4_CON	Construction as % of GDP (dev. fm optimum)	3% of GDP + the annual GDP growth rate	0.04
ii.4_MIL	Military expenses as % of GDP (dev. fm optimum)	-For low- and lower-middle-income countries: 1% of GDP; -For upper-, middle- and high-income countries: 2% of GDP; -For CHN, GBR, RUS, USA, DEU, FRA: 3% of GDP; -For Israel and Middle East countries: 5% of GDP	0.014
ii.4_UNI	Unionization rate (dev. fm optimum)	10%	15.16
iii.7_GPS	Expenditure on general public services as % of GDP (dev. fm optimum)	4%	3.156
iii.8_DCT	Corporate tax rate (dev. fm optimum)	24%	7.111
iii.8_DTR	Tax revenue as % of GDP (dev. fm optimum)	11%	6.199
iv.11_DMA	M&A as % of investment—3 yrs. rolling average (dev. fm. Optimum)	8%	0.107
iv.12_LFR	Labor force participation ratio—male vs female	100	20.09

Standard Deviation of raw indicator values (‘Std.Dev.’) is rounded to three decimals. The rationale for each conceptual optimum is provided in Chapter “5.2 Indicator Table: Definitions and Rationale” in Casas and Cozzi (2021)

### ${\varvec{X}}_{5}$—***Normalisation Scheme***

Input factor $X_{5}$ selects one of three normalisation options. Irrespective of the chosen scheme, prior to normalisation, a logarithmic transformation is applied to indicator datasets: firstly, if Pearson’s second coefficient of skewedness exceeds unity, which indicates strong skewedness (Fávero & Belfiore, 2019, p. 63); and secondly, if that indicator is not already based on an existing index. This is to improve the distribution of the data and thus yield more meaningful indicator scores. In total, 21 of the 107 EQx2021 Indicators are based on a logarithmic transformation. Furthermore, final indicator scores are adjusted for polarity, so that—consistently across all indicators—a score close to 100 indicates a high level of elite quality, and a value close to 0 represents a low level of elite quality.

#### EQx-normalisation

The first option represents the EQx normalisation scheme. First, a z-score standardisation is applied:

$$ I_{q,c}^{z} = \frac{{x_{{{\text{q}},{\text{c}}}} - mean\left( {x_{{\text{q}}} } \right)}}{{sd\left( {x_{{\text{q}}} } \right)}} $$

where $x_{{{\text{q}},{\text{c}}}}$ indicates the value of indicator $q$ of country $c$, and $I_{{{\text{q}},{\text{c}}}}$ denotes the standardised value. This yields values with a mean of zero and standard deviation of one. Next, if necessary, outliers are winsorised to fall within a [− 2, + 2] interval. The resulting values are then rescaled to fall within a [0,100] interval, applying:

$$ I_{q,c}^{{}} = \left( {\frac{{I_{q,c}^{z} }}{4} + 0.5} \right) * 100 $$

#### MinMax

The second option standardises values according to the Min–Max procedure, applying:

$$ I_{q,c}^{m} = \frac{{x_{{{\text{q}},{\text{c}}}} - min_{{\text{c}}} \left( {x_{{\text{q}}} } \right) }}{{max_{{\text{c}}} \left( {x_{{\text{q}}} } \right) - min_{{\text{c}}} \left( {x_{{\text{q}}} } \right)}} $$

This yields values with a range [0,1]. Next, values are rescaled to fall within a [0,100] interval, applying:

$$ I_{q,c}^{{}} = I_{q,c}^{m} *100 $$

### ${\varvec{X}}_{6}$—***Weighting Scheme***

Table 6 below describes the baseline weighting scheme applied to the EQx2021 (column (1)), as well as 9 alternative weighting schemes (columns (2) to (10)) used for the MCEQx.

**Table 6:** Alternative weighting schemes considered for input factor $X_{6}$

	EQx (1)	Altern. 1 (2)	Altern. 2 (3)	Altern. 3 (4)	Altern. 4 (5)	Altern. 5 (6)	Altern. 6 (7)	Altern. 7 (8)	Altern. 8 (9)	Altern. 9 (10)
Indicator weight within pillar	BAP	Equal	✓	Equal	Equal	Equal	Equally weighted indicators without any other structure; individual final indicator weight:$1/107$	✓	✓	✓
Pillar weight within index area	BAP	✓	Equal	Equal	Equal	Equal		✓	✓	✓
Index area weight within sub-index										
Political Power	$0.\overline{3}$	✓	✓	✓	Equal	Equal		✓	✓	Equal
Economic Power	$0.\overline{6}$	✓	✓	✓	Equal	Equal		✓	✓	Equal
Political Value	$0.\overline{3}$	✓	✓	✓	Equal	Equal		✓	✓	Equal
Economic Value	$0.\overline{6}$	✓	✓	✓	Equal	Equal		✓	✓	Equal
Sub-indices										
Power	$0.\overline{3}$	✓	✓	✓	✓	Equal		0.25	Equal	Equal
Value	$0.\overline{6}$	✓	✓	✓	✓	Equal		0.75	Equal	Equal

‘BAP’ indicates weights determined by the Budget Allocation Process, ‘equal’ indicates equal weighting within the respective level. The check mark indicates that the baseline weighting scheme is not changed

### ${\varvec{X}}_{7}$—***Aggregation Scheme***

Input factor $X_{7}$ selects one of two aggregation options at the sub-index level: either linear or geometric. These are the two most commonly adopted techniques. They differ with respect to the extent of compensability they imply between the aggregated index elements. While linear aggregation implies constant and full compensability between index elements, compensability is lower for an index element with low values, in the case of geometric aggregation (Munda, 2012, p. 338; OECD, 2008, p. 33).

At the sub-index level, the EQx score $Y_{{\text{c}}}$ for country $c, c = 1,...M$ is calculated as a function of the country’s sub-index scores $I_{{{\text{s}},{\text{c}}}}$ ($S = 2$), and the within EQx sub-index weights, $w_{s}$. Sub-index Power has a weight of 1/3, and Value a weight of 2/3. When using linear aggregation, index scores result from the following formula:

$$ Y_{{\text{c}}} = \mathop \sum \limits_{s = 1}^{S} w_{s} I_{{{\text{s}},{\text{c}}}} $$

while geometric aggregation applies:

$$ Y_{{\text{c}}} = \mathop \prod \limits_{s = 1}^{S} I_{{{\text{s}},{\text{c}}}}^{{w_{s} }} $$

## A2 Detailed Results of Uncertainty Analysis

Figure 4 below illustrates the frequency a country is placed in each tenner ranking class according to the MCEQx. Frequencies less than 5% are omitted. Countries are ordered and numbered according to their EQx2021 rank.

## Footnotes

1 For more details on the index methodology, the reader is referred to Casas et al. (2020) and Chapter 2 in Casas and Cozzi (2021).

2 Note that a measurement error can generally be decomposed into a systematic error (bias) and a random error (variance). Input factor ${\mathrm{X}}_{1}$ only accounts for the latter form of error, since we do not expect the data to be generally biased.

3 The generation of Sobol’ quasi-random number sequences and the estimation of first and total-order Sobol’ indices are implemented with the statistical software R and the recently published *sensobol* package (Puy, Lo Piano, et al., 2021a).
